# Induction of exportin-5 expression during melanoma development supports the cellular behavior of human malignant melanoma cells

**DOI:** 10.18632/oncotarget.11410

**Published:** 2016-08-19

**Authors:** Corinna Anna Ott, Lisa Linck, Elisabeth Kremmer, Gunter Meister, Anja Katrin Bosserhoff

**Affiliations:** ^1^ Institute of Biochemistry, Emil-Fischer-Zentrum, Friedrich-Alexander University Erlangen-Nürnberg, 91054 Erlangen, Germany; ^2^ Biochemistry Center Regensburg (BZR), Laboratory for RNA Biology, University of Regensburg, 093053 Regensburg, Germany; ^3^ Institute of Molecular Immunology, Helmholtz Center Munich, German Research Center for Environmental Health (GmbH), 81377 Munich, Germany; ^4^ Comprehensive Cancer Center Erlangen-EMN, Friedrich-Alexander University Erlangen-Nürnberg, 91054 Erlangen, Germany

**Keywords:** malignant melanoma, microRNA, XPO5, miR-SNP, mRNA stability

## Abstract

Regulation of gene expression via microRNAs is known to promote the development of many types of cancer. In melanoma, miRNAs are globally up-regulated, and alterations of miRNA-processing enzymes have already been identified. However, mis-regulation of miRNA transport has not been analyzed in melanoma yet. We hypothesized that alterations in miRNA transport disrupt miRNA processing. Therefore, we investigated whether the pre-miRNA transporter Exportin-5 (XPO5) was involved in altered miRNA maturation and functional consequences in melanoma. We found that XPO5 is significantly over-expressed in melanoma compared with melanocytes. We showed enhanced XPO5 mRNA stability in melanoma cell lines which likely contributes to up-regulated XPO5 protein expression. In addition, we identified MEK signaling as a regulator of XPO5 expression in melanoma. Knockdown of XPO5 expression in melanoma cells led to decreased mature miRNA levels and drastic functional changes. Our data revealed that aberrant XPO5 expression is important for the maturation of miRNAs and the malignant behavior of melanoma cells. We suggest that the high abundance of XPO5 in melanoma leads to enhanced survival, proliferation and metastasis and thereby supports the aggressiveness of melanoma.

## INTRODUCTION

Malignant melanoma, a tumor derived from melanocytes, is the most aggressive form of skin cancer, and there is no cure for patients with advanced melanomas [1]. MicroRNAs (miRNAs) are small non-coding RNA molecules that suppress gene expression on the post-transcriptional level by targeting mRNAs [2, 3]. They are involved in the development and progression of a variety of human cancer types, including melanoma [4–7]. The biogenesis of miRNAs is a multi-step process. First, the primary miRNA transcript (pri-miRNA) is cleaved by Drosha and its cofactor DGCR8 [8–14]. The precursor-miRNA (pre-miRNA) hairpin is then transported into the cytoplasm via exportin-5 (XPO5) [15–18]. In the cytoplasm, the pre-miRNA is processed by Dicer and its cofactor TRBP to a double stranded miRNA/miRNA* intermediate [19,20]. Finally, one strand of the duplex is degraded, whereas the functional miRNA strand is incorporated into the RNA-induced silencing complex (RISC) where it directly binds to a member of the Argonaute (Ago) protein family [21,22]. The miRNA-containing RISC is then guided to the target mRNA, which is deadenylated, degraded or translationally repressed [23–25].

Some miRNAs are already known to have the important function of inhibiting the expression of specific genes in malignant melanoma, leading to melanoma formation and progression (reviewed in [6, 17, 18]). In contrast to other cancers, where most miRNAs are down-regulated compared to their expression in healthy tissue [28,29], miRNAs are commonly up-regulated in malignant melanoma compared with healthy skin [26, 27, 30] As alterations in miRNA transport could be a reason for the elevated miRNA levels in melanoma, we aimed to investigate whether the pre-miRNA transporter XPO5 contributes to the development and/or progression of malignant melanoma by influencing miRNA transport and maturation.

## RESULTS & DISCUSSION

### Enhanced XPO5 protein expression and mRNA stability in malignant melanoma

**Figure 1:**
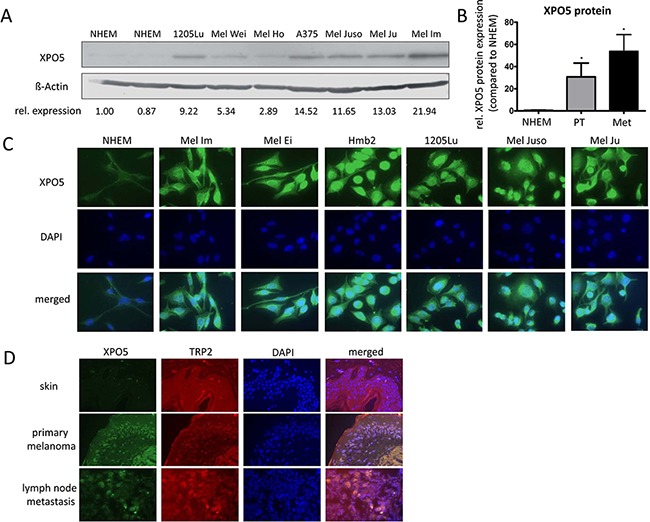
XPO5 protein expression in NHEMs, melanoma cell lines and tissue samples **A.** Western blot results showing XPO5 protein expression in normal human epidermal melanocytes (NHEMs) and primary (PT) and metastatic (Met) melanoma cell lines. ß-Actin was used as a loading control. The relative densitometric quantifications of XPO5 protein expression are indicated under the blot and show a strong induction of XPO5 protein expression in melanoma cells compared with NHEMs. **B.** Relative densitometric quantification of three independent Western blot analyses compared with ß-Actin. XPO5 expression in NHEMs was set as 1. **C.** Protein expression of XPO5 (as detected by immunofluorescence staining) was enhanced in melanoma cell lines compared with NHEMs. **D.** Protein expression of XPO5 (as detected by immunofluorescence staining) was enhanced in primary melanoma tissue and tissue of a lymph node metastasis compared with healthy skin. TRP2 staining was used to stain melanocytes.

We determined the level of XPO5 protein expression in melanoma via Western blot analysis using samples from normal human epidermal melanocytes (NHEMs) and the following melanoma cell lines: 1205Lu, Mel Wei, Mel Ho, A375, Mel Juso, Mel Ju and Mel Im (Figure 1A). XPO5 protein expression was significantly up-regulated in the tested primary tissues (PT) and in the metastatic (Met) melanoma cell lines compared with NHEMs (Figure 1B). Immunofluorescence staining for XPO5 in the NHEMs and the melanoma cell lines (Mel Im, Mel Ei, Hmb2, 1205Lu, Mel Juso and Mel Ju) confirmed the strong over-expression of XPO5 protein in the melanoma cell lines compared with that in the NHEMs (Figure 1C). As expected, XPO5 protein was found equally distributed in the nucleus as well as in the cytoplasm, illustrating the nucleo-cytoplasmic shuttling of the protein. Immunofluorescence analysis of skin, primary melanoma and lymph node metastases with antibodies against XPO5 showed that the XPO5 protein expression level was elevated in primary and metastatic melanomas from human melanoma patient samples compared with healthy skin (Figure 1D).

We show, for the first time, that XPO5 protein levels are significantly increased in malignant melanoma compared with NHEMs. Together with the finding that AGO2 protein expression is decreased in malignant melanoma [31] and the fact that miRNAs are highly abundant in melanoma in contrast to healthy skin and melanocytes [30], our discovery of elevated XPO5 protein levels in melanoma supports the assumption that miRNAs compete for AGO protein binding and that only the most efficiently expressed miRNAs result in mRNA regulation.

**Figure 2:**
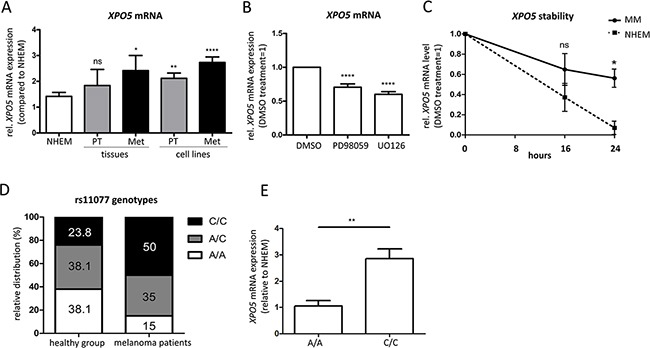
Elevated XPO5 mRNA expression and stability in malignant melanoma **A.** qRT-PCRs showed an increase in expression of XPO5 mRNA during melanoma progression in primary and metastatic melanoma tissues (n=4) and cell lines (n=13) compared with NHEMs (n=15). **B.** XPO5 mRNA expression decreased after treatment with the MEK inhibitors PD98059 and U0126 in comparison to DMSO-treated cells (n=10). **C.** The stability of XPO5 mRNA was determined by qRT-PCR after treatment of NHEMs and melanoma cell lines (MM) with alpha-amanitin for 0, 16 and 24 hours. The remaining XPO5 mRNA level in the melanoma cell lines (solid line) was significantly different to that of the NHEMs (dashed line) after the 24 h treatment (n=3). **D.** The relative distribution of the rs11077 genotypes in melanoma patients (n=20) versus the healthy control group (n-21). In the control group, the values are equivalent to the distribution in the Caucasian population. In melanoma patients, half of the patients had the C/C variant SNP (50%). **E.** Relative XPO5 mRNA expression compared to NHEM in the homozygous rs11077 genotypes A/A and C/C.

In contrast to protein expression levels, the expression of XPO5 mRNA has already been analyzed in melanoma. Sand et al. (2012) screened melanoma patient samples using TaqMan RT-PCR, but did not find significant differences in XPO5 mRNA expression [32]. Similarly, we performed qRT-PCR analyses to determine XPO5 mRNA levels in melanoma tissues of patients and melanoma cell lines (Figure 2A). We showed that in primary melanoma tissues (PT) and metastatic melanoma tissues (Met), XPO5 mRNA expression is slightly increased compared with NHEMs. This increase of XPO5 mRNA expression was significant for the metastatic melanoma tissues but not for the primary melanoma tissues. In the melanoma cell lines, a slight but significant elevation of XPO5 mRNA expression was observed in cells derived from primary tumors and those derived from metastases. In addition to the study by Sand et al., which compared mRNA expression levels of XPO5 in melanoma tissue and samples of benign nevi, we used melanocytes – the cells of origin for melanoma – for comparison [32]. When screening heterogeneous nevi samples, other cell types besides melanocytes, such as keratinocytes and fibroblasts, were tested.

As approximately 50% of melanomas harbor BRAF mutations, the Raf/MEK/ERK signaling pathway is constitutively activated, leading to enhanced ERK activity [33–36]. Moreover, inhibition of MEK inhibits melanoma cell proliferation and invasion and induces apoptosis [37–39]. Due to the ability of ERK to activate transcription factors and thereby regulate gene expression, we tested whether the enhanced XPO5 expression in melanoma could be caused by mis-regulated MEK/ERK signaling by treating the melanoma cell lines Mel Im and Hmb2 with the MEK inhibitors PD98059 and U0126 for 48 hours. A slight but significant reduction of XPO5 mRNA expression was observed after treatment with the inhibitors (Figure 2B), hinting to the potential regulation of XPO5 expression via MEK/ERK signaling in malignant melanoma.

Because we expected stronger effects on the mRNA expression of XPO5 after MEK/ERK inhibition, we analyzed XPO5 mRNA stability in melanoma cells in comparison to melanocytes. We quantified XPO5 mRNA expression in melanoma cell lines and NHEMs that were treated with α-amanitin for 0, 16 and 24 hours to inhibit RNA polymerases II and III (Figure 2C). We observed that after 16 hours, a slight decrease in mRNA levels was found in the NHEMs compared to the melanoma cell lines (not significant), while after 24 hours, the levels of XPO5 were significantly lower in the NHEMs. The mRNA expression of XPO5 in melanocytes dropped to only 10% compared with untreated NHEMs. However, in melanoma cells, 60% of the XPO5 mRNA could still be detected after RNA polymerase inhibition for 24 hours, hinting that XPO5 mRNA was more stable in malignant melanomas than in the NHEMs.

As XPO5 mRNA is more stable in malignant melanoma cells than in melanocytes, we tested whether the SNP rs11077 could be the reason for altered XPO5 mRNA stability. In the last few years, a role of this SNP has been described in many different diseases, including its association with the worst chance of survival in cancer [40–44]. To screen for SNP variants, we sequenced the DNA and cDNA (depending on availability) of healthy samples (n=21) compared to probes of melanoma patients (n=20) and found that healthy donors mainly had the A/C or A/A variant (38.1% each), while the SNP variant (C/C) was present in only 23.8% of the donors (Figure 2D). Interestingly, in melanoma patients, a shift of genotype distribution could be observed. While the amount of the heterozygous variant stayed nearly the same (35.0%), the homozygous C genotype was detected in 50.0% of the melanoma patient samples. The proportion of the A/A variant dropped to 15.0% in melanoma patients. This SNP leads to a loss of the miR-617 binding site in the XPO5 3′UTR [45]. As the expression of miR-617 was equally detected in the NHEMs and the melanoma cell lines (own miRNA array data, data not shown), we assume that due to the loss of the miR-617 binding site in the C/C-variant, less degradation of XPO5 via miR-617 can occur. We could also find a correlation between the SNP genotype and XPO5 mRNA expression (Figure 2E). Cell lines and patient samples with the C/C variant displayed a significant higher XPO5 mRNA expression than those with the homozygous A genotype. In conclusion, we determined that elevated XPO5 mRNA stability due to a loss of the miR-617 binding site via the SNP rs11077 is one reason for enhanced XPO5 expression in melanoma.

### Regulation of XPO5 expression

XPO5 protein expression is highly upregulated in melanoma cells compared with melanocytes, while mRNA levels are only mildly increased. We, therefore, hypothesized that altered XPO5 mRNA expression cannot be the only reason for enhanced XPO5 protein levels in malignant melanoma. Therefore, we examined other factors which play an important role in melanoma and could lead to strong differences in protein expression. To test whether the decrease in XPO5 protein levels in NHEMs could be a result of proteasomal degradation, we treated NHEMs with the proteasome inhibitor MG132. Subsequent Western blot analyses revealed no effect on XPO5 protein expression after MG132 treatment with three different concentrations compared to the control (DMSO)-treated NHEMs (Supplementary Figure S1A). The effect of MG132 on the proteasome could be demonstrated previously [46]. The metastatic melanoma cell line Mel Im served as an internal positive control for XPO5 protein expression.

The transcription factor NFκB is strongly upregulated in malignant melanoma, leading to an elevated expression of oncogenes such as N-Cadherin and Cyclin D1 [47,48]. We therefore wanted to determine whether NFκB up-regulation and activity could be responsible for the high expression of XPO5 in malignant melanoma. We transduced the melanoma cell lines Mel Ei and Mel Im with an Ad5IĸB adenovirus expressing the NFκB super-repressor, to reduce NFκB transcription factor activity and analyzed XPO5 protein expression via Western blot. Efficient inhibition of NFκB was demonstrated previously [47]. As the inhibition of NFκB did not decrease XPO5 expression in both cell lines used (Supplementary Figure S1B), we excluded elevated NFκB activity as a reason for the up-regulated XPO5 expression in malignant melanoma.

Next, we analyzed whether the protein kinase C (PKC) might be involved in XPO5 expression by treating cells with the PKC inhibitor staurosporine (ST). Functionality of ST could be confirmed by CRE luciferase assay (Supplementary Figure S1C) However, the Western blot analysis displayed no differences in XPO5 protein expression in Mel Juso and HTZ19 melanoma cells (Supplementary Figure S1D), leading to the conclusion that XPO5 expression is not activated by PKC.

In 2013, Iwasaki et al. published that XPO5 expression is induced via a phosphoinositide 3-kinase (PI3K)-dependent post-transcriptional mechanism in embryonic mouse fibroblasts [49]. Therefore, we investigated whether PI3K signaling was the cause for enhanced XPO5 expression in melanoma (Supplementary Figure S1E). However, the inhibition of PI3K via treatment with LY-294002 or Wortmannin decreased the downstream Akt phosphorylation but did not lower XPO5 protein levels in melanoma. Inhibition of p-Akt could also be seen after serum starvation of Mel Im cells, with no effect on XPO5 expression. Re-feeding the cells with fetal calf serum (FCS) re-induced Akt phosphorylation, but XPO5 expression levels were not affected. Overall, these results show that XPO5 expression is not influenced by the PI3K signaling pathway in malignant melanoma.

**Figure 3:**
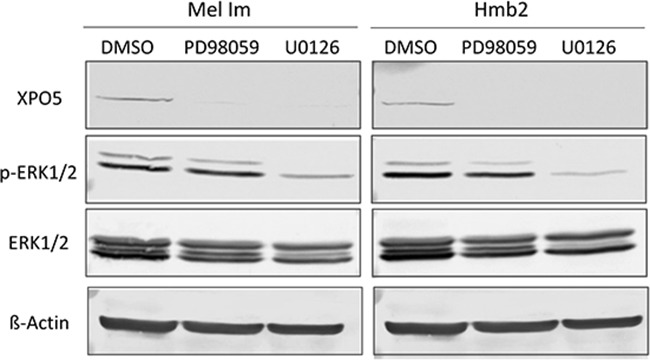
Analysis of potential regulators of XPO5 protein expression Western blot analyses after the treatment of the melanoma cell lines Mel Im and Hmb2 with the MEK inhibitors PD98059 and U0126 showed a strong downregulation of XPO5 expression. The p-ERK1/2 staining reflects inhibition of ERK phosphorylation by U0126 treatment in both cell lines compared with the control treatment (DMSO). ERK1/2 antibody staining was used to show overall ERK level. ß-Actin was analyzed in the same protein samples on a second blot (due to similar molecular weights) and served as a loading control.

As the inhibition of the MEK/ERK signaling pathway decreased XPO5 mRNA expression (Figure 2B), we subsequently tested its effect on XPO5 protein expression. As seen in the Western blot analysis in Figure 3, a massive reduction of XPO5 protein expression was observed after treatment with both PD98059 and U0126 in both cell lines used. However, ERK phosphorylation was only slightly reduced by PD98059 treatment while U0126 led to a massive drop in p-ERK. U0126 is an effective inhibitor of MEK1 and MEK2 activity, whereas PD98059 prevents the activation of only MEK1 [50,51]. As both inhibitors equally decreased XPO5 levels, we assume that MEK1 participates in XPO5 regulation. In accordance with the elevated activity of MEK in melanoma, this result supports the conclusion that XPO5 expression is induced via a yet unknown MEK-dependent, ERK-independent signaling mechanism in malignant melanoma.

**Figure 4:**
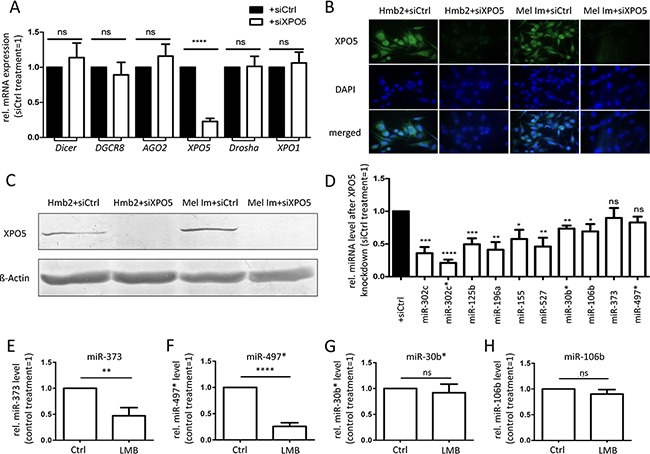
Consequences of siRNA-induced XPO5 downregulation in the metastatic melanoma cell lines Mel Im and Hmb2 **A.** Relative expression of genes involved in miRNA processing and mRNA transport. A significant knockdown of XPO5 in melanoma cell lines did not alter the gene expression of Dicer, DGCR8, AGO2, Drosha or XPO1 compared with control treatment (siCtrl) (n=7). **B.** Immunofluorescence staining and **C.** Western blot analysis of XPO5 showed a strong downregulation of XPO5in Mel Im and Hmb2 cells after siXPO5 treatment. **D.** Relative levels of mature miRNAs after XPO5 knockdown compared with siCtrl (n=4). Except for miR-373 and miR-497*, the levels of all tested miRNAs were significantly lower compared with those of the siCtrl treatment. **E-H.** Relative miRNA levels after XPO1 inhibition (n=4). The levels of (E) miR-373 and (F) miR-497* were significantly lower after XPO1 inhibition via leptomycin B (LMB) compared with control-treated cells (Ctrl). The levels of (G) miR-30b* and (H) miR-106b were not affected by XPO1 inhibition.

Next, we analyzed the functional relevance of altered XPO5 expression. Therefore, we established siXPO5 transfection experiments. To exclude the off-target effects of transfection we also tested the mRNA expression of other genes involved in miRNA processing or (RNA) export. After transfection with siRNA pools against XPO5 the mean remaining mRNA expression of XPO5 was 18.1% in Mel Im cells and 27.8% in Hmb2 cells (Figure 4A). However, the mRNA expression levels of Dicer, DGCR8, AGO2, and Drosha, as well as the RNA transporter Exportin-1 (XPO1), were not significantly altered by siXPO5 treatment. Via XPO5 immunofluorescence and Western blot analysis, we verified a strong XPO5 knockdown after siRNA treatment in both cell lines used. (Figure 4B and 4C). After the knockdown of XPO5 via siRNA transfection, miRNA levels were analyzed. The levels of the miRNAs miR-302c, miR-302c*, miR-125b, miR-196a, miR-155, miR-527, miR-30b*, miR-106b, miR-373 and miR-497* were chosen to exemplify the effect of XPO5 knockdown on the cytoplasmic level of miRNAs. Except for miR-373 and miR-497*, the mature miRNA levels of all tested miRNAs were significantly lower after XPO5 down-regulation (Figure 4D). This result is consistent with the findings of Lund et al., who showed the down-regulation of miRNA levels after XPO5 knockdown in HeLa cells [52]. Similarly, it has already been shown that the level of miRNAs can be directly induced by over-expression of XPO5 in the fibroblasts of embryonic mice [49]. We showed, for the first time, that miRNA levels correlate with XPO5 expression in malignant melanoma.

**Figure 5:**
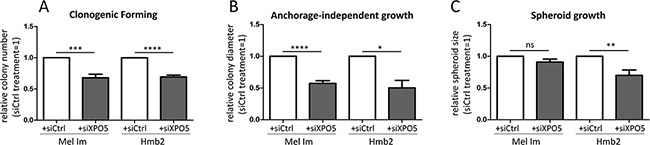
Functional consequences of XPO5 knockdown in melanoma cell lines **A.** siRNA-induced XPO5 knockdown in Mel Im and Hmb2 significantly decreased the number of colonies formed in the clonogenic forming assay compared with that of siCtrl-treated cells (n=7). **B.** XPO5 knockdown resulted in significantly smaller colonies of Mel Im and Hmb2 cells in the anchorage-independent growth assay using soft agar (n=3). **C.** Spheroid growth of siXPO5-treated Hmb2 cells was significantly decreased compared with siCtrl-treated cells (n=5).

To test whether XPO1 can act as an alternative transporter for miRNAs in melanoma, we treated melanoma cells with the XPO1 inhibitor leptomycin B (LMB; Figures 4E-4H). The cytoplasmic levels of miR-373 and miR-497*, which were not decreased by XPO5 knockdown via siRNA treatment, were significantly decreased by XPO1 inhibition via LMB treatment (Figure 4E and 4F), indicating that XPO1 acts as an alternative nucleo-cytoplasmic transporter for these miRNAs. The levels of miR-30b* and miR-106b, which were significantly decreased by XPO5 knockdown, were not influenced by XPO1 inhibition (Figure 4G and 4H), showing that XPO5 is their main transporter. Interestingly, it has been previously shown that microprocessor-independent miRNAs also use XPO1 for nuclear-cytoplasmic shuttling in HEK and HCT116 cells [53–55]. To analyze the functional consequences of enhanced XPO5 expression in melanoma cells, clonogenic assays with Mel Im and Hmb2 cells after siXPO5 transfection were performed. In both cell lines, the number of cell clones built from single-seeded cells was significantly lower in cells in which XPO5 was knocked down than in control transfected cells (Figure 5A). In addition, anchorage-independent growth assays using soft-agar plated wells showed that the ability to build attachment-free 3-dimensional colonies was reduced in siXPO5 treated melanoma cells (Figure 5B). Moreover, Hmb2 melanoma cells with downregulated XPO5 levels showed a significantly reduced capacity to form multicellular spheroids (Figure 5C). As the knockdown of XPO5 had significant functional effects on the cells tested, we assume that XPO5 induces miRNA levels and thereby (indirectly) stimulates cellular characteristics such as proliferation, survival and aggressiveness, as reflected in our functional assays.

### Functional consequences of XPO5 over-expression in melanocyte-like cell clones

**Figure 6:**
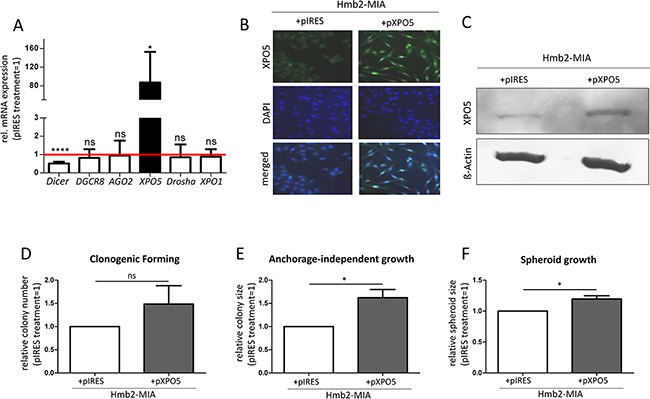
Over-expression of XPO5 in MIA-deficient Hmb2 cell clones and its functional consequences **A.** Significant over-expression of XPO5 in Hmb2-MIA cells did not alter the gene expression of DGCR8, AGO2, Drosha or XPO1 compared with that in the control treatment (pIRES) but significantly decreased Dicer gene expression (n=3). **B.** Immunofluorescence staining and **C.** Western blot analysis of XPO5 showed strong XPO5 over-expression in Hmb2-MIA cells after pXPO5 treatment compared with pIRES transfection. **D-F.** Functional consequences of XPO5 over-expression in Hmb2-MIA cells. (D) XPO5 over-expression in Hmb2-MIA increased the number of colonies formed in the clonogenic forming assay compared with pIRES-treated cells, but the increase was not statistically significant (n=4). (E) XPO5 over-expression resulted in significantly larger colonies of in the anchorage-independent growth assay (n=3). (F) Spheroid growth of pXPO5 transfected Hmb2-MIA cells was significantly increased compared with that of pIRES-treated cells (n=4).

In parallel to the XPO5 knockdown experiments in metastatic melanoma cell lines, MIA-negative melanocyte-like cell clones (Hmb2-MIA; [56]) were transfected with the control plasmid pIRES and the XPO5 plasmid (pXPO5) to determine the effects of XPO5 over-expression on melanocyte-like cells. We achieved an average 72.9-fold induction of XPO5 mRNA expression and no significant alterations of DGCR8, AGO2, Drosha or XPO1 mRNA levels after the over-expression of XPO5 by transfecting the cells with pXPO5 (Figure 6A). Interestingly, induction of XPO5 in this cell line induced a significant down-regulation of Dicer mRNA levels. In contrast, we did not detect alterations in Dicer mRNA expression after XPO5 knockdown, which is surprising because in 2011, Bennasser et al. reported down-regulated Dicer mRNA levels after siXPO5 in HeLa cells [57].

As seen in the immunofluorescence results in Figure 6B and in the Western blot (Figure 6C), XPO5 protein expression was considerably elevated after transfection with the XPO5 expression plasmid. Clonogenic forming assays with pXPO5 transfected cell clones showed a higher number of colonies compared with Hmb2-MIA cells transfected with the control plasmid (Figure 6D). Moreover, XPO5 over-expressing cells built visibly larger colonies (data not shown), indicating that in addition to the elevated clone-building capability, XPO5 over-expression also had an effect on 3-dimensional proliferation. Furthermore, anchorage-independent growth assays demonstrated that XPO5 over-expression resulted in the growth of significantly larger colonies (Figure 6E). This assay confirmed the enhanced proliferation of the melanocyte-like cell clones after the induction of XPO5 expression compared with control plasmid-transfected Hmb2-MIA. In spheroid assays, pXPO5-transfected Hmb2-MIA cells displayed significantly larger cell clusters than control plasmid-transfected cells, demonstrating that XPO5 was involved in the potential of cells to build 3-dimensional cell clusters and to proliferate within the spheroid (Figure 6F). Similar to our 3-dimensional assays after XPO5 knockdown (Figure 5A–5C), the functional effects after XPO5 over-expression on cellular characteristics underline the hypothesis that mis-regulated XPO5 protein expression contributes to melanoma cell survival, proliferation and malignancy.

The results of this study showed that the pre-miRNA transporter XPO5 is up-regulated in malignant melanoma as a consequence of de-regulated MEK signaling and enhanced XPO5 mRNA stability. This high expression of XPO5 leads to enhanced 3-dimensional proliferation, clone-building ability, and cell clustering by promoting miRNA maturation and inducing mature miRNA levels. These findings highlight the importance of precisely adjusted miRNA levels and, moreover, miRNA machinery protein expression in malignant melanoma.

## MATERIALS AND METHODS

### Cell culture and tissue samples

The melanoma cell lines Mel Juso, Mel Im, Mel Ju, Mel Wei, Mel Ho, Mel Ei, A375, HTZ19, 1205Lu, and Hmb2, as well as normal human epidermal melanocytes (NHEMs), were previously described. The Mel Juso, Mel Wei, Mel Ho and Mel Ei cell lines were derived from primary cutaneous melanomas, and the Mel Im, Mel Ju, 1205Lu, A375, HTZ19 and HMB2 cell lines were derived from metastases of malignant melanomas. The cells were maintained in DMEM supplemented with penicillin (400 units/ml), streptomycin (50 mg/ml), and 10% FCS (Sigma-Aldrich, Steinheim, Germany), and they were incubated in a humidified atmosphere containing 8% CO_2_ at 37°C. They were split at a ratio of 1:5 every 3 days. NHEMs (PromoCell, Heidelberg, Germany) were derived from neonatal skin. Hmb2-MIA is a cell clone resembling melanocytes that was generated in our laboratory [58]. Once per week, this cell line was treated with geneticin (2 mg/ml) to ensure selection. Tissue samples of snap-frozen melanoma primary tumors and melanoma metastases were obtained from patient tissue collection at the Institute of Pathology, University of Regensburg, Germany. Sampling and handling of patient material was carried out in accordance with the ethical principles of the Declaration of Helsinki. Cell culture starvation experiments were performed using DMEM with penicillin, streptomycin and 2% FCS.

### Transfection of cells with XPO5-siRNA, XPO5 plasmid and IκB adenovirus transduction

For siRNA transfection, 1.5×10^5^ melanoma cells per well were seeded in six-well plates and transfected with 50 pmol of XPO5 siRNA (siXPO5) or negative control siRNA (siCtrl) pool stocks (siTOOLs Biotech, Planegg/Martinsried, Germany [59]) using Lipofectamine RNAiMAX reagent (Life Technologies, Darmstadt, Germany). The cells were incubated at 37°C until use. For XPO5 plasmid transfection, 2×10^5^ Hmb2-MIA cells per well were seeded and transfected after 4 h with 1 μg of XPO5 expression plasmid (pXPO5) or control plasmid (pIRES) using Lipofectamine Plus reagent according to the manufacturer's instructions (Life Technologies). The generation of the IκB adenovirus (Ad5IκB) has been previously described [60]. To prevent the phosphorylation of inducible IκB, the adenoviral vector bears a mutant form of the NFκB super-repressor IκB where serines 32 and 36 are replaced by alanine (S32A/S36A). The transduction of cells with Ad5IκB and the control virus Ad5LacZ were performed as previously described [47,60].

### Treatment of cells with inhibitors

For the treatment of melanoma cells with inhibitors, 2×10^5^ melanoma cells per well were seeded in six-well plates. After 4 h, the cells were treated with 20 mM of the PI3 kinase inhibitor LY-294002, 20 mM of Wortmannin (both Sigma-Aldrich, St Louis, MO, USA) or a control substance for 16 h. For the inhibition of XPO1, cells were incubated with 10 ng/ml of leptomycin B (LMB, Sigma-Aldrich) for 20 h. For MEK inhibition, cells were treated with 10 μM PD98059 or 10 μM U0126 (Merck Millipore, Billerica, MA, USA) for 48 h. RNA polymerase was inhibited using 5 μM α-amanitin (AppliChem, Darmstadt, Germany). Treatment with MG132 (Merck Millipore) at final concentrations of 5, 10 and 20 μM for 24 hours was used to inhibit the proteasome. For protein kinase inhibition, cells were treated with 100 μM staurosporine (Merck Millipore) for 16 hours.

### Transfection and CRE luciferase assay

In all, 2×10^5^ melanoma cells per well were seeded in six-well plates and transfected with 0.5 μg of CRE reporter constructs using Lipofectamine LTX (Invitrogen). For co-transfection experiments, in addition to the reporter construct, 0.5 μg of expression plasmid or empty vector was transfected using Lipofectamine Plus (Invitrogen). The cells were lysed 18 hours after transfection with 1x Passive Lysis Buffer (Promega, Mannheim, Germany) and luciferase activity was determined. All samples were co-transfected with 0.5 μg of a pRL-TK plasmid (Promega) to normalize transfection efficiency. Renilla luciferase activity was measured by a luminometric assay (Dual-Luciferase Reporter Assay; Promega).

### Total RNA and microRNA isolation and reverse transcription

Before RNA isolation, melanoma tissue samples in tubes containing 1.4 mm ceramic beads (Peqlab, Erlangen, Germany) and lysis buffer were disrupted using a Precellys 24 tissue homogenizer (Bertin Technologies, Montigny-le-Bretonneux, France). Total cellular RNA was isolated from cultured cells and homogenized tissues using the E.Z.N.A. Total RNA Kit I (Omega Bio-Tek, VWR, Darmstadt, Germany) according to the manufacturer's instructions. Complementary DNAs of total RNA were generated by reverse transcriptase reaction using the Super Script II Reverse Transcriptase Kit (Life Technologies).

MiRNAs were isolated using the MiRNeasy Mini Kit (Qiagen, Hilden, Germany). Reverse transcription of miRNAs was performed using the miScript II RT Kit (Qiagen) according to the manufacturer's instructions.

### Analysis of mRNA and miRNA expression via quantitative PCR

Quantitative real time-PCR of total RNA was performed using a LightCycler 480 (Roche, Mannheim, Germany). The PCR reaction consisted of 500 ng of cDNA template, 0.5 μl (20 μM) of the forward and reverse primers and 10 μl of LightCycler 480 SYBR Green I Master Mix (Roche) in a total volume of 20 μl, and the following PCR program was used: 30 s 95°C (initial denaturation) 20°C/s temperature transition rate up to 95°C for 15 s, 10 s annealing at 60°C, 20 s 72°C, 10 s acquisition mode single, repeated for 45 times (amplification). Annealing and melting temperatures were optimized for each primer set (Table 1). Gene expression was normalized to the expression of the housekeeping gene ß-Actin.

**Table 1:** Oligonucleotide sequences

Gene	forward sequence (5′-3′)	reverse sequence (5′-3′)
AGO2	GTCTCTGAAGGCCAGTTCCA	ATAGAGGCCTCACGGATGG
ß-Actin	CTACGTCGCCCTGGACTTCGAGC	GATGGAGCCGCCGATCCACACGG
DGCR8	AGCAGGCGGAGTCCGAGAGG	TGTGTGCTTGCCACACGCCA
Dicer	GCCCTGTGCCCTACTCGGGA	GTGCTGCCGCGGGTCTTCAT
Drosha	GGACCTGCGCGAAGTCTGGC	AGGTCTTGGTGCGAAGCGCC
XPO1	GAGCAAGTAGGACCAGCGAA	TCCCAGGGGAATCCAGTTCA
XPO5	TGGCCACAGAGGTCACCCCC	GGGGCGCAGTGCCTCGTAT
XPO5-genomic	CTTGGACCACTCCAACTCCC	GTACTGTGGCTCTTGTGCCT

For the detection of the SNP genotypes, PCR reactions were performed with the genomic XPO5 primer using Fast Start Taq Polymerase (Roche) and the following PCR program: activation for 5 min at 95°C and 40 cycles of 30 s at 95°C, 30 s at 62°C, and 90 s at 72°C. After PEG-precipitation, the DNA was sequenced (Microsynth, Balgach, Switzerland).

qRT-PCR of reverse transcribed miRNAs was performed on a LightCycler 2.0 (Roche). Each 20 μl reaction consisted of 1 μl of template cDNA, 2 μl of miScript Primer Assays (Qiagen), 2 μl of miScript Universal Primer (Qiagen) and 10 μl of miScript SYBR Green. The following PCR program was used: 15 min at 95°C and 40 cycles of 15 s at 94°C, 30 s at 55°C, and 30 s at 70°C. U6 rRNA was used for miRNA expression normalization. All PCR reactions were evaluated by melting curve analysis.

### Antibody generation

A peptide comprising amino acids psktdspsceysrfd from human XPO5 protein was synthesized and coupled to ovalbumin (OVA) or bovine serum albumin (BSS)(PSL GmbH, Heidelberg, Germany). Lou/c rats were immunized subcutaneously and intraperitoneally with a mixture of 50 μg OVA-peptide, 5 nmol CPG oligonucleotide (Tib Molbiol, Berlin), 500 μl PBS and 500 μl incomplete Freund's adjuvant. A boost without adjuvant was given six weeks after the primary injection. Fusion was performed using standard procedures. Supernatants were tested in a differential ELISA with the BSA-XPO5 peptide and an irrelevant OVA-peptide on ELISA plates. MAbs that reacted specifically with the XPO5 (EX5) peptide were further analyzed in Western blot. The hybridoma cells of one XPO5-reactive supernatant (EX5 4E1) was cloned by limiting dilution. Tissue culture supernatant of EX5 4E1 (rat IgG1/κ) was used in this study.

### Western blot analysis

Melanoma tissue samples in tubes containing 1.4 mm ceramic beads and 200 μl of radio-immune precipitation assay (RIPA) buffer (Roche) were disrupted using a Precellys 24 homogenizer. Cells were resuspended in 200 μl of RIPA buffer and lysed for 15 min at 4°C. Insoluble fragments were removed by centrifugation (13 000 rpm, 10 min, 4°C), and the supernatant was stored at −20°C. RIPA lysates (40 μg per lane) were loaded and separated on 10% SDS polyacrylamide gels and subsequently blotted onto a PVDF membrane (Bio-Rad, Berkeley, CA, USA). After blocking for 1 h with 3% BSA/PBS (anti-ß-Actin or anti-p-Akt) or 5% non-fat dry milk/TBS-T (anti-XPO5, anti-p-ERK1/2, anti-ERK1/2), the membrane was incubated over night at 4°C with 1:20 of rat anti-XPO5 (EX5 4E1) in blocking solution, 1:5000 of mouse anti-ß-Actin (Sigma-Aldrich) in PBS, and 1:5000 of either rabbit anti-p-ERK1/2, rabbit anti-ERK1/2 or rabbit anti-pAkt (Cell Signaling, Cambridge, UK) in 5% BSA/TBS-T. After it was washed three times with TBS-T, the membrane was incubated for 1 h with a 1:3000 dilution of an alkaline phosphate-coupled secondary anti-mouse (Chemicon, Hofheim, Germany), a 1:5000 dilution of an anti-rat alkaline phosphatase antibody (Sigma-Aldrich) or a 1:4000 dilution of an anti-rabbit alkaline phosphatase antibody (Cell Signaling) in TBS-T, followed by three more washes in TBS-T. Finally, the immunoreactions were visualized by NBT/BCIP staining (Sigma-Aldrich).

### Immunofluorescence staining and analysis

For immunofluorescence assays, cells were seeded in chamber slides, washed with PBS, fixed with pre-chilled acetone for 10 min at −20°C, permeabilized using 0.1% TritonX-100/PBS for 5 min, washed again and blocked for 1 h with 1% BSA/PBS. Subsequently, the cells were incubated with a 1:10 dilution of anti-XPO5 antibody (EX5 4E1) or anti-TRP2 (Sigma) in 1% BSA/PBS overnight at 4°C. After they were washed with PBS three times, the cells were incubated with a 1:50 dilution of FITC-conjugated anti-rat immunoglobulin for 1 h, washed again with PBS and mounted with VECTASHIELD Hard Set Mounting Medium containing DAPI (Vector Laboratories, Burlingame, CA, USA). Images were taken using immunofluorescence microscopy with an Axio Imager Zeiss Z1 fluorescence microscope (AxioVision Rel. 4.6.3, Carl Zeiss AG, Oberkochen, Germany).

### Clonogenic assay

Clonogenic assays were performed in duplicate 4 days after the first transfection (transfections on day 0 and 4) of melanoma cells as previously described [61]. In brief, transfected cells were seeded at a low density in six-well plates (100 and 200 cells per well) and incubated at 37°C for 6 days. Then, the cells were rinsed with PBS, fixed with 6% v/v glutaraldehyde and stained with crystal violet. Colony numbers were counted manually.

### Anchorage-independent growth assay

Anchorage-independent growth assays were performed four days after the first transfection (transfection on days 0 and 3). Briefly, six-well plates were coated with 2 ml of 0.5% soft agar containing DMEM, non-essential amino acids and FCS. After the agar had set, 1000 and 2000 cells in 400 μl were mixed with 600 μl of ground agar mixture and plated on top of the ground agar. Experiments were performed in duplicates. Plates were incubated at 37°C for 9 days. Then, colony sizes were measured using a light microscope.

### Spheroid assay

Cells were used 4 days after the first transfection (transfections on days 0 and 3). For the spheroid assay, 100 μl of the cell suspension containing 4000 cells was added to each well of a 96-well plate coated with 1% agarose. Per transfection experiment, 20-30 replicates were made. After 3 days of incubation, spheroid sizes were measured using a light microscope.

### Image analyses

For densitometric measurements of Western blot assay bands, the free software Fiji, downloaded under http://www.fiji.sc, was used following the User Guide [62]. Semi-quantitative analyses were made by measuring bands in relation to the respective ß-Actin band.

### Statistical analyses

Results are expressed as the mean ± standard deviation. Comparison between groups was made using Student's unpaired t-test. p-values < 0.05 were considered statistically significant (ns, not significant, *p < 0.05, **p < 0.01, ***p < 0.001, ***p < 0.0001). All calculations were performed using GraphPad Prism Software (GraphPad Software, Inc., San Diego, USA).

## SUPPLEMENTARY FIGURE


