# Supplementary material for: Induction of exportin-5 expression during melanoma development supports the cellular behavior of human malignant melanoma cells
Source: Oncotarget. 2016 Aug 19;7(38):62292–304. doi: 10.18632/oncotarget.11410 (PMC5308727; doi:10.18632/oncotarget.11410)
Supplement: Supplementary file 1 [file oncotarget-07-62292-s001.pdf]

Induction of exportin-5 expression during melanoma development supports the cellular behavior of human malignant melanoma cells

SUPPLEMENTARY FIGURE

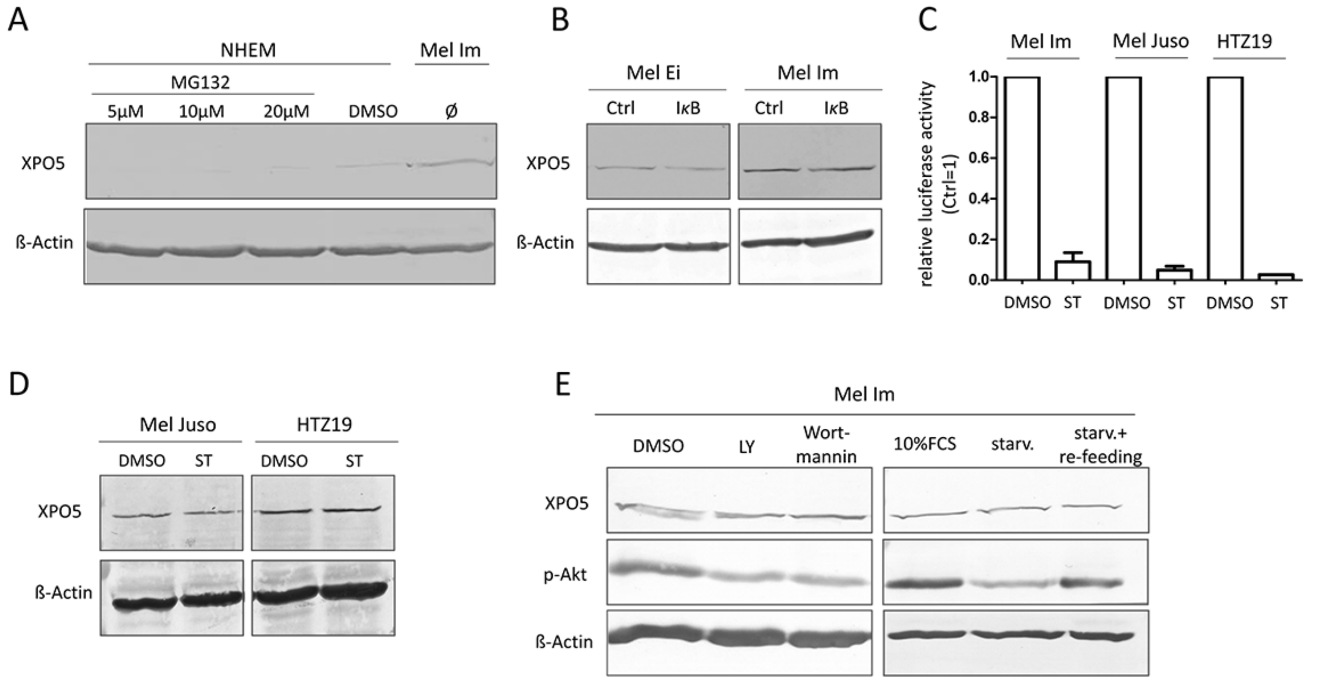

Supplementary Figure S1: Analysis of factors and signaling pathways not involved in regulation of XPO5 expression. **A.** No elevation in XPO5 protein expression was observed after proteasome inhibition via MG132 in NHEMs compared with DMSO-treated cells. Mel Im cells were used as a positive control and displayed high levels of XPO5 protein. **B.** Mel Ei and Mel Im melanoma cells were transduced with the NFκB-inhibitory Ad5IκB adenovirus (IκB) or the control adenovirus Ad5LacZ (Ctrl) for 48 h. Western blot analysis showed no influence of NFκB inhibition on XPO5 protein expression compared with control transduction. ß-Actin was used as a loading control. **C, D.** Inhibition of protein kinase C via staurosporine (ST), demonstrated by decreased CRE luciferase activity, did not affect XPO5 protein expression. **E.** After inhibition of Akt phosphorylation via PI3K inhibitor treatment, no changes in XPO5 protein expression could be observed. Serum starvation (starv) of melanoma cells led to decreased Akt phosphorylation, while refeeding the cells with serum-containing medium induced phosphorylation of Akt (starvation and re-feeding). During the starvation and refeeding of cells, XPO5 expression levels did not changed.
